# Automatic detection of lucky bamboo nodes based on Improved YOLOv7

**DOI:** 10.3389/fpls.2025.1604514

**Published:** 2025-07-17

**Authors:** Jing Zhang, Ruoling Deng, Chengzhi Cai, Erpeng Zou, Haitao Liu, Mingxin Hou, Xinzhi Chen, Huamin Lin, Zhenye Wei

**Affiliations:** ^1^ School of Mechanical Engineering, Guangdong Ocean University, Zhanjiang, Guangdong, China; ^2^ Guangdong Engineering Technology Research Center of Ocean Equipment and Manufacturing, Guangdong Ocean University, Zhanjiang, Guangdong, China; ^3^ Guangdong Provincial Key Laboratory of Intelligent Equipment for South China Sea Marine Ranching, Guangdong Ocean University, Zhanjiang, Guangdong, China; ^4^ College of Mathematics and Computer Science, Guangdong Ocean University, Zhanjiang, Guangdong, China

**Keywords:** lucky bamboo, handicraft, convolutional neural network, YOLOv7, object detection, bamboo node

## Abstract

**Introduction:**

The detection of lucky bamboo (*Dracaena sanderiana*) nodes is a critical prerequisite for machining bamboo into high-value handicrafts. Current manual detection methods are inefficient, labor-intensive, and error-prone, necessitating an automated solution.

**Methods:**

This study proposes an improved YOLOv7-based model for real-time, precise bamboo node detection. The model integrates a Squeeze-and-Excitation (SE) attention mechanism into the feature extraction network to enhance target localization and introduces a Weighted Intersection over Union (WIoU) loss function to optimize bounding box regression. A dataset of 2,000 annotated images (augmented from 1,000 originals) was constructed, covering diverse environmental conditions (e.g., blurred backgrounds, occlusions). Training was conducted on a server with an RTX 4090 GPU using PyTorch.

**Results:**

The proposed model achieved a 97.6% mAP@0.5, significantly outperforming the original YOLOv7 (83.4% mAP) by 14.2%, while maintaining the same inference speed (100.18 FPS). Compared to state-of-the-art alternatives, our model demonstrated superior efficiency. It showed 41.5% and 153% higher FPS than YOLOv11 (70.8 FPS) and YOLOv12 (39.54 FPS), respectively. Despite marginally lower mAP (≤1.3%) versus these models, the balanced trade-off between accuracy and speed makes it more suitable for industrial deployment. Robustness tests under challenging conditions (e.g., low light, occlusions) further validated its reliability, with consistent confidence scores across scenarios.

**Discussion:**

The proposed method significantly improves detection accuracy and efficiency, offering a viable tool for industrial applications in smart agriculture and handicraft production. Future work will address limitations in detecting nodes obscured by mottled patterns or severe occlusions by expanding label categories during training.

## 1 Introduction

Lucky bamboo (*Dracaena sanderiana*) is a potted ornamental plant with excellent ornamental value (Chen, 2012; Akinlabi et al., 2017; Gergel and Turner, 2017). Traditional lucky bamboo is only used for flower arrangement or direct potting, with low added value (Rout et al., 2006; van Dam et al., 2018; Abdel-Rahman et al., 2020). Processing lucky bamboo into handicrafts can substantially increase its ornamental value, which is deeply loved by the public and has a large demand in the international market (Sharma et al., 2014; Amin and Mujeeb, 2019; Liu et al., 2019). Since the processing of lucky bamboo is cutting the lucky bamboo according to the bamboo nodes to meet different requirements, identifying the lucky bamboo nodes is the first and most crucial step in processing lucky bamboo. However, the existing methods of identifying lucky bamboo nodes still mainly rely on manual work, which has the disadvantages of low efficiency, high labour cost, and prone to errors. Therefore, studying a method that can automatically recognize lucky bamboo nodes with high efficiency and precision is imperative.

In recent years, traditional image processing methods have been widely used to identify bamboo-related fields. Juyal P et al. used methods such as logistic regression, support vector machine, naive Bayesian, random forest, convolutional neural network and ResNet to conduct a comparative analysis, and finally could accurately identify five common bamboos (Juyal et al., 2020). Watanabe used convolutional neural networks (CNN) to identify Japanese bamboo forest areas through Google satellite images, with an overall recognition accuracy of 93.7% (Watanabe et al., 2020). Kumar conducted research on bamboo leaf disease detection and developed a program to automatically recognize bamboo leaf diseases based on image processing and CNN (Kumar et al., 2022). Ziwei Wang used a residual neural network, original dataset, and MixUp dataset to optimize the traditional algorithm CNN to further improve the classification ability of bamboo species (Wang et al., 2022). Pankaja used Fourier descriptors to extract bamboo leaf features and used the Bayes classifier to identify bamboo leaves with an accuracy of 88.03% (Pankaja and Thippeswamy, 2017).

Existing target detection algorithms can be roughly divided into two categories. The first category is the two-stage R-CNN (He et al., 2017) series of algorithms based on region proposal, such as R-CNN, Fast R-CNN (Li et al., 2017), Faster R-CNN (Salvador et al., 2016), etc. The position of the object frame usually needs to be found first and then the category of the object frame will be determined by this algorithm. Although this type of method has high recognition accuracy, it takes a long time to calculate and is not suitable for real-time detection. The second category is the one-stage algorithm (Tian et al., 2022) represented by YOLO (Redmon, 2016) and SSD (Liu et al., 2016). This type of algorithm takes regression as the core, omitting the region proposal link of the two-stage algorithm, directly distinguishing specific categories and returning the bounding box (Redmon and Farhadi, 2017). Shilan Hong used optimized YOLOv4 to model the detection of bamboo shoots and proposed a classification and screening strategy to track each bamboo shoot (Hong et al., 2022). The experimental results showed that the average relative error and variance of the number of bamboo shoots were 1.28% and 0.016%, respectively, and the average relative error and variance of the corresponding pixel height results were -0.39% and 0.02%, respectively. The advantage of this method is that it can perform real-time detection, which is beneficial to improving the recognition efficiency of bamboo nodes. However, there is much room for improvement in the detection accuracy and robustness of this method, and its detection ability for small targets is also relatively poor. The current target detection algorithm cannot take into account both detection accuracy and timeliness, and the detection effect for small target units such as bamboo nodes is relatively poor.

To solve the above problems, this paper proposed a method for real-time and precise detection of luck bamboo nodes based on the improved model of YOLOv7. Firstly, an attention mechanism was introduced into the feature extraction network to enhance effective feature information and suppress invalid information. This can help the model locate and identify the lucky bamboo nodes faster and more accurately. Then, WIoU was introduced in the loss value calculation to optimize the bounding box regression process of the bamboo node through a dynamic weighting mechanism, thereby improving the model’s detection ability for complex scenes and small bamboo nodes.

The main objectives of this research were to (a) construct the image dataset of lucky bamboo nodes using the data augmentation method, (b) establish the lucky bamboo node detection model using the improved YOLOv7, and (c) evaluate the detection stability and accuracy of the proposed method.

## 2 Materials and methods

### 2.1 Dataset construction

**Figure 1:**
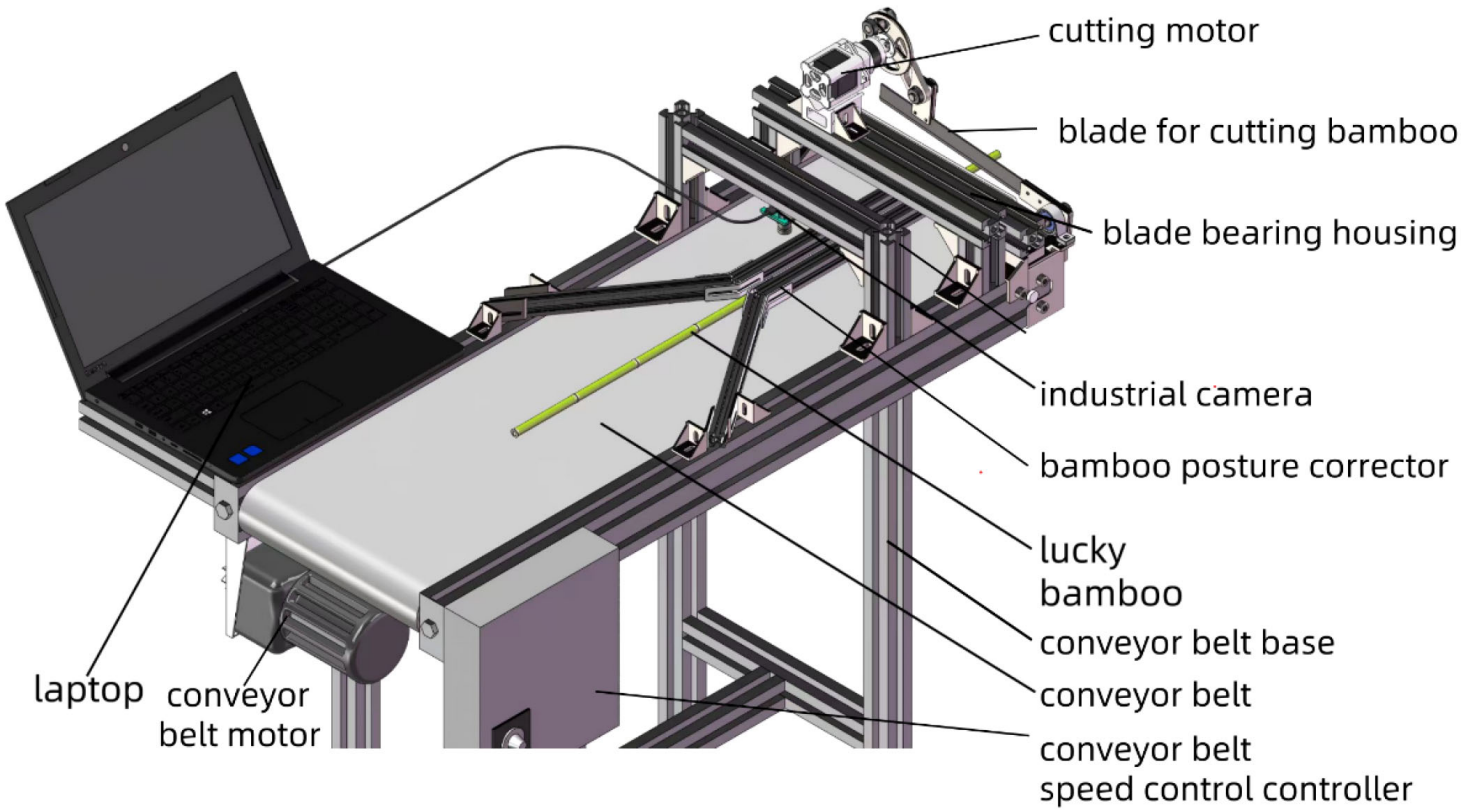
Acquisition equipment of lucky bamboo images.

The image collecting equipment used in this study is shown in **Figure 1**. The equipment consisted of an USB industrial camera, an assembly line workbench, and a laptop. The assembly line workbench was equipped with conveyor belt, a conveyor belt motor, conveyor belt speed control controller, bamboo posture corrector, a cutting motor, a blade for cutting bamboo, a blade bearing housing, and conveyor belt base. The industrial camera was mounted on the tube, parallel to the conveyor belt.

**Figure 2:**
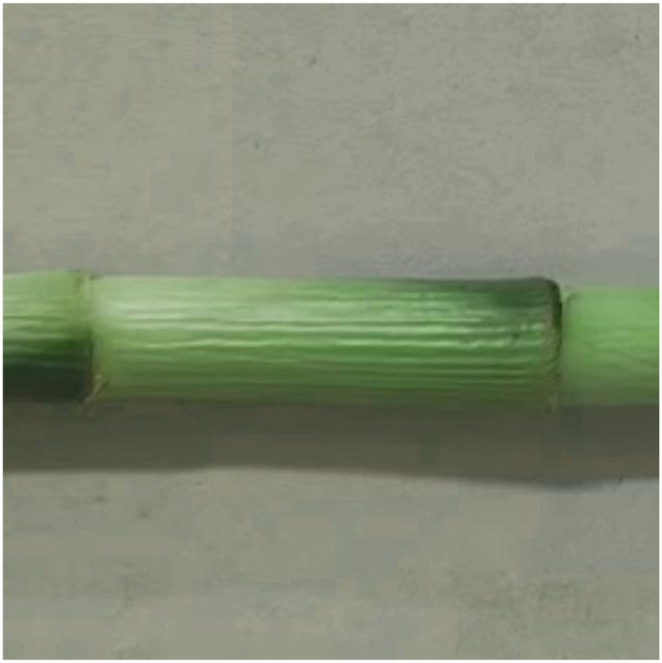
Images of lucky bamboo.

The lucky bamboo samples were planted in the processing base of Fugui Horticultural Farm in Mazhang District, Zhanjiang City, Guangdong Province, China. The images of lucky bamboo were collected using USB industrial camera connected to a laptop on November 1, 2023. The focal length of the camera lens is 3.6 mm. Specifically, the lucky bamboo was placed on the horizontal conveyor belt and then photographed at a vertical height of 50 mm from the lucky bamboo. Since the original lucky bamboo image had a resolution of 2 million pixels, which was too large and will increase the amount of calculation, OpenCV in Python was used to process the original image. Finally, the original images were compressed to 640×640 pixels, with a horizontal and vertical resolutions of 96 dpi. Among them, the lucky bamboo image was stored in JPG format with a size of 32 MB. In the end, a total of 1,000 lucky bamboo images were collected, as shown in **Figure 2**.

**Figure 3:**
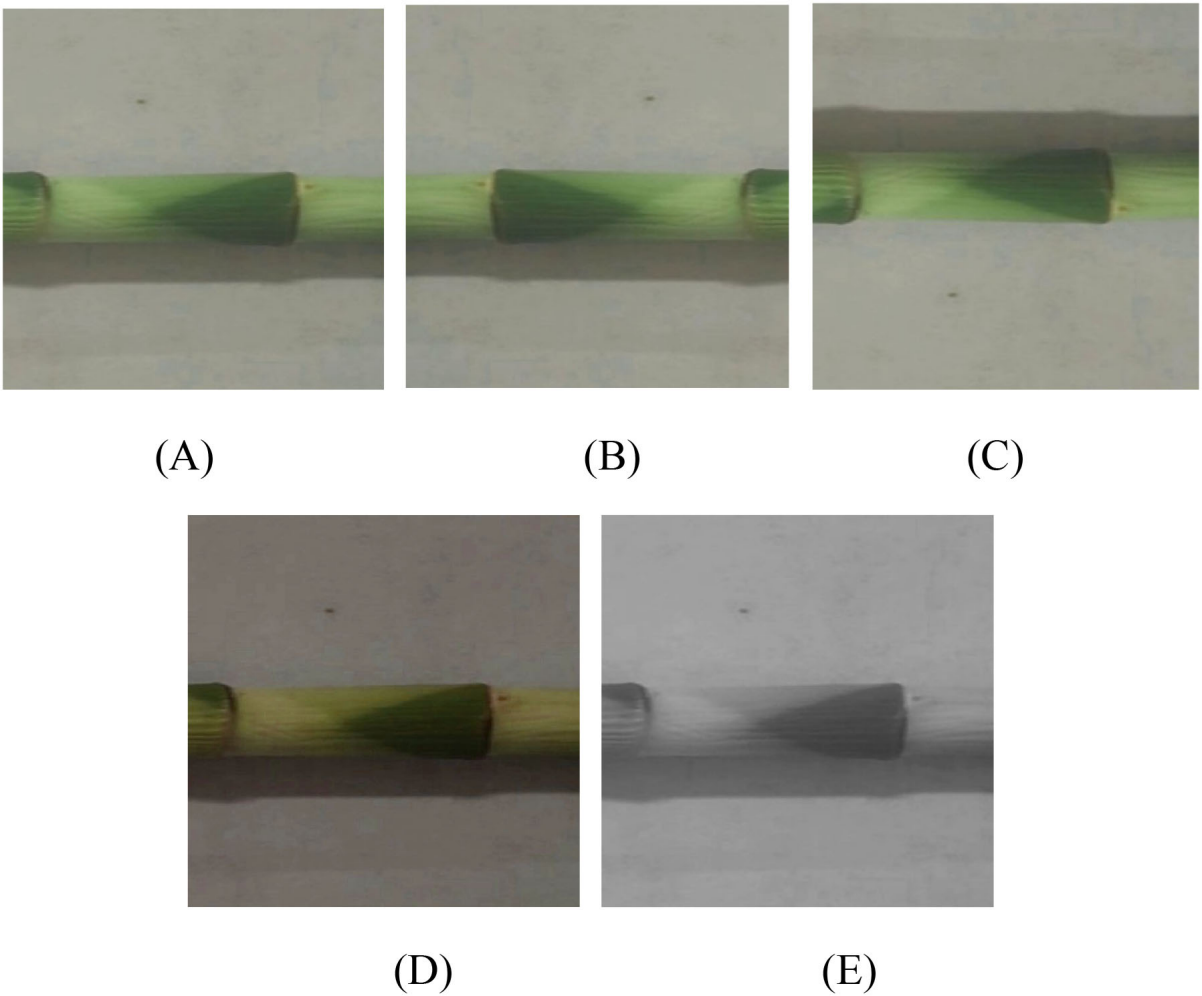
Data augmentation of luck bamboo nodes. **(A)** original image; **(B)** random horizontal flip, **(C)** random vertical flip, **(D)** color jitter, **(E)** random grayscale.

To improve the generalization ability and robustness of the model and avoid model overfitting, the transforms module based on the Pytorch deep learning framework was used to perform data augmentation and expansion on the collected images (**Figure 3A**) (Cubuk et al., 2019). Since two major data enhancement methods (geometric transformation (flip) and color space perturbation (jitter + grayscale) had been widely proven to effectively improve the generalization ability of the model (Shorten and Khoshgoftaar, 2019). Therefore, the lucky bamboo images were performed random horizontal flip (**Figure 3B**), random vertical flip (**Figure 3C**), and color jitter (**Figure 3D**). Also, the RandomGrayscale function was used to convert the image to grayscale with a probability of 2.5% (**Figure 3E**), allowing the model to learn image features without color information and enhance the detection capabilities in complex scenes. After data enhancement, a total of 5000 images were obtained. Finally, 2000 representative images were obtained for constructing the lucky bamboo dataset. Also, the bamboo dataset was randomly divided into training set, verification set, and testing set in a ratio of 7:2:1 for model training and testing.

**Figure 4:**
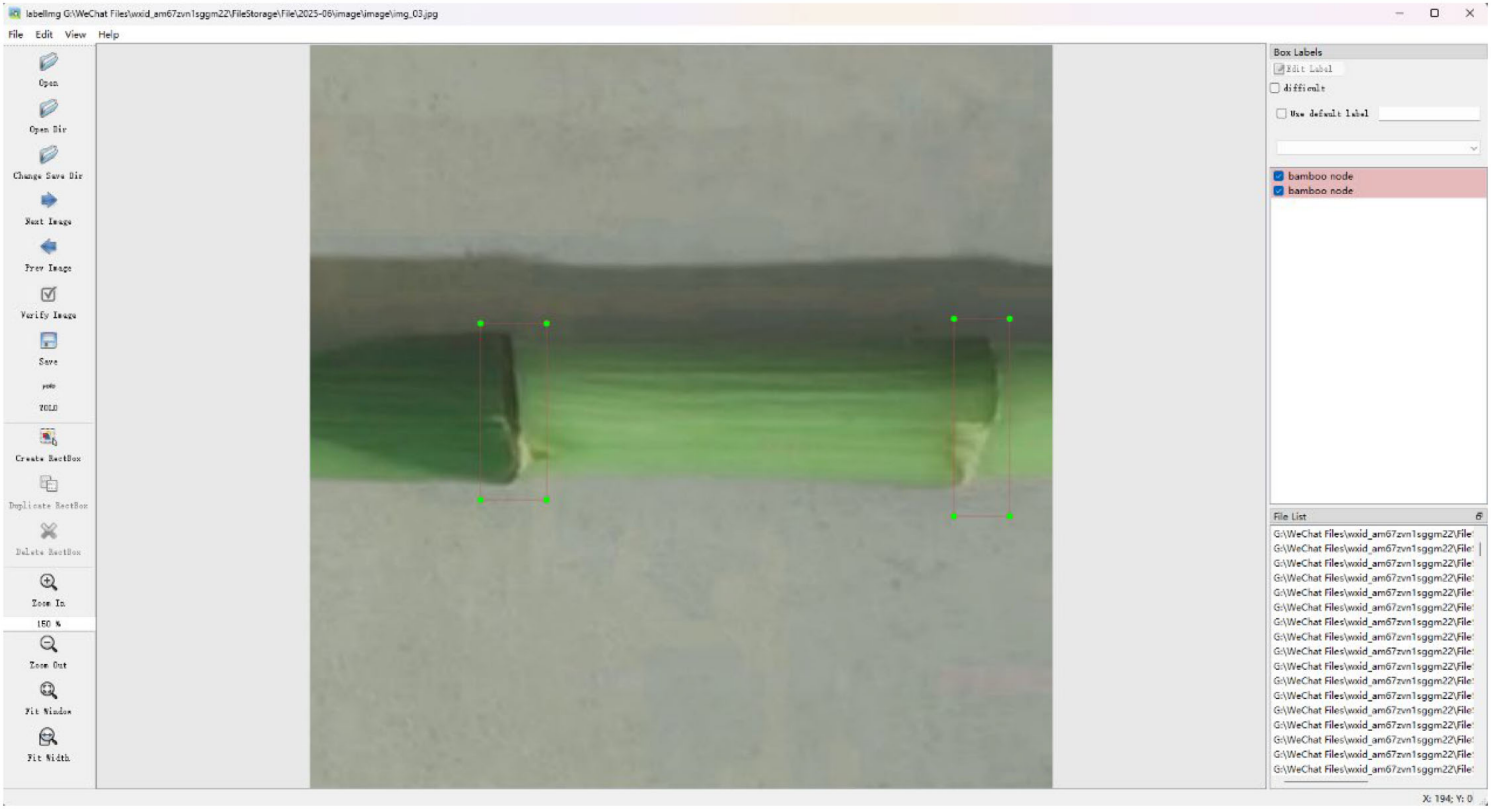
The image annotation of lucky bamboo nodes (Darrenl, 2017).

To enable the model to accurately locate bamboo nodes, the open-source software LabelImg was used to manually annotate all the lucky bamboo images after data augmentation (**Figure 4**) (Darrenl, 2017). In this study, the content of annotation was each node of lucky bamboo, that was, the collected location coordinate information. After labeling, all data were saved in the Pascal VOC dataset format. The annotation diagram is shown in **Figure 4**. Among them, the green frame represented the position of the bamboo nodes of lucky bamboo in the image.

### 2.2 Bamboo node detection method

#### 2.2.1 Advantage of YOLOv7 model

YOLO (You Only Look Once) was a deep learning algorithm for real-time object detection that only required one forward propagation through a given neural network to detect all objects in the image (Redmon, 2016). This gave the YOLO algorithm an advantage over other algorithms in terms of speed, making it one of the most famous detection algorithms to date.

YOLO divided the image into multiple small grids and predicted multiple bounding boxes in each grid, as well as the object categories within each bounding box. YOLO used a single neural network to predict all bounding boxes and classes in an image, instead of using multiple neural networks to predict each bounding box. The advantage of YOLO was that it can run in real time and can detect more objects. YOLOv7 (Wang et al., 2023) was optimized on the basis of YOLOv4 (Bochkovskiy et al., 2020), training a better model with less training time. The input layer of YOLOv7 supported image enhancement (such as Mosaic) and adaptive anchor box calculation. Its backbone network used CSPDarknet53 and enhanced feature extraction capabilities by using CSPNet. Also, the Neck module in YOLOv7 combined Path Aggregation Network (PANet) and Spatial Pyramid Pooling (SPP) modules, which can better handle multi-scale features and enhance the accuracy of the model.

**Figure 5:**
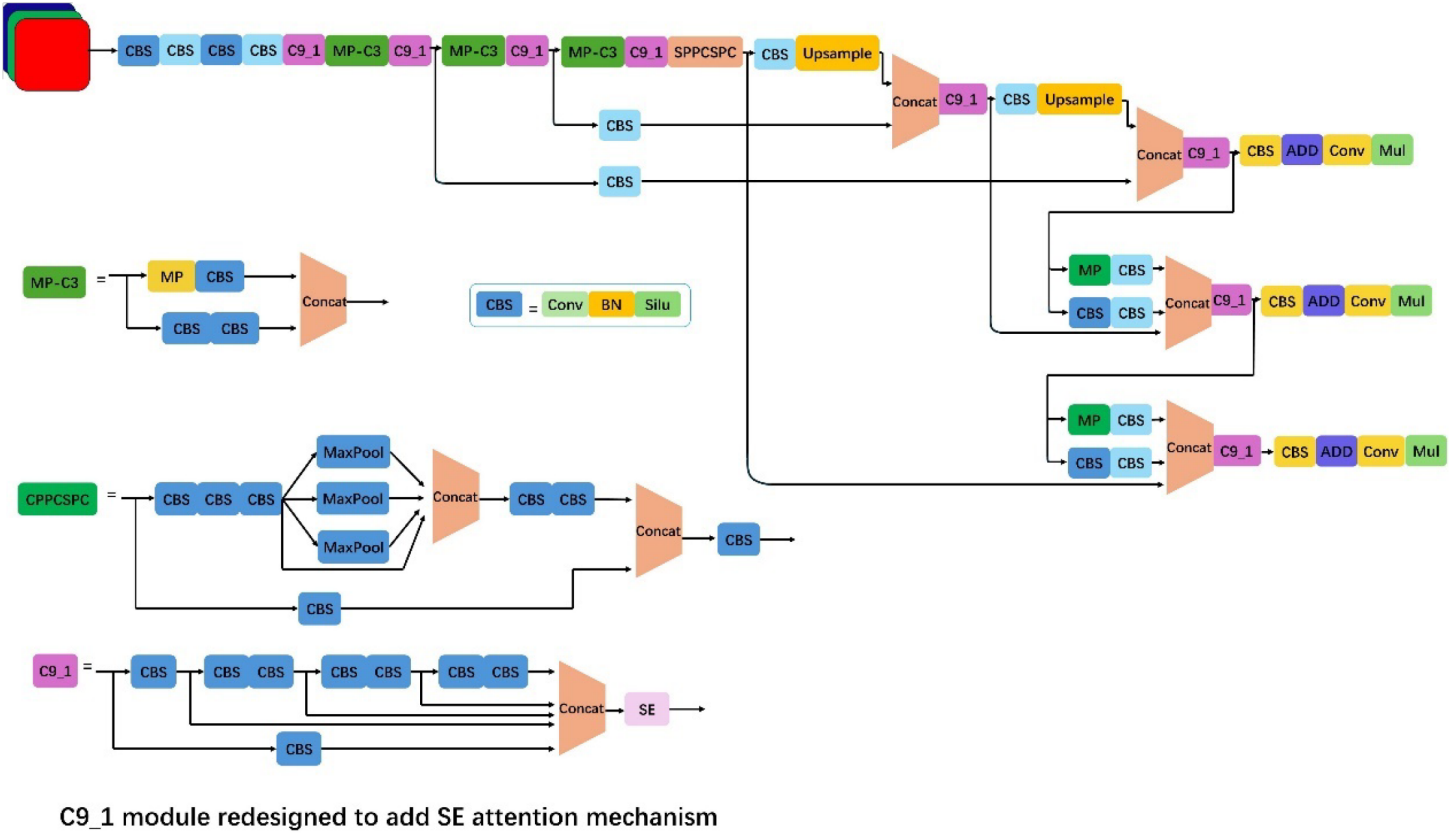
Architecture of the improved YOLOv7.

The algorithm mainly consisted of an input terminal, a feature extraction network, a feature fusion network, and an output terminal (**Figure 5**). Compared with YOLOv4, YOLOv7 employed a Focus operation that sampled the original image at double intervals in both horizontal and vertical directions. This reduced FLOPs value and computational complexity, thereby improving detection speed. Additionally, in the feature extraction module, YOLOv7 replaced the original CSP module with the C3 module. This modification enhanced training speed, reduced gradient redundancy, and improved learning efficiency. For network input processing, YOLOv3 (Farhadi and Redmon, 2018) and YOLOv4 required executing a separate program to compute initial anchor boxes when training on different datasets. In contrast, YOLOv7 integrated this functionality directly into the framework, enabling adaptive calculation of optimal anchor boxes for each training scenario. Also, unlike two-stage algorithms (e.g., Faster R-CNN), YOLOv7 eliminated the computationally intensive feature extraction and region proposal steps, significantly reducing inference time. Although the accuracy of YOLOv7 was slightly lower than that of Faster RCNN, its detection speed was faster and supported real-time detection. Therefore, YOLOv7 was selected as the basic framework for detecting lucky bamboo nodes.

#### 2.2.2 Problems caused by using YOLOv7 model

YOLOv7 was currently the best-engineered generation object detection model of the YOLO series. It achieved real-time image processing speeds while maintaining accuracy compared to state-of-the-art models. Therefore, it was widely used in real-time vision applications. However, YOLOv7 still had some shortcomings in the application environment of lucky bamboo node detection in this paper, which were mainly reflected in the following aspects.

(a) The regression idea of the YOLO algorithm was to divide the image into S×S grids, that was, each grid could only predict at most one object. Consequently, when multiple objects occupied the same grid, the algorithm’s detection performance degraded significantly, often failing to identify all objects.
(b) The original network employed the Generalized Intersection over Union (GLoU) (Rezatofighi et al., 2019) loss function for bounding box regression. However, GIoU demonstrated limited effectiveness for small object detection, as it failed to explicitly incorporate object scale considerations. Furthermore, this loss function may induce a bias toward predicting larger bounding boxes, ultimately compromising detection accuracy.

#### 2.2.3 Construction of bamboo node detection model

In response to the problems raised above, the following improvements were made to the YOLOv7 original network in this paper.

(a) Adding the SE attention mechanism (Hu et al., 2018; Niu et al., 2021) can make the network pay more attention to the bamboo nodes to be detected, thereby improving the model accuracy. The SE attention mechanism adaptively recalibrated channel-weight feature responses through its Squeeze and Excitation operations, selectively enhancing discriminative features. This mechanism enhanced feature representation accuracy by adaptively amplifying salient features while suppression irrelevant channel responses. Furthermore, the SE module enhanced model performance in complex scenarios through learned channel-weight adaptation, dynamically optimizing feature attention to improve both robustness and detection accuracy.
(b) To enhance model detection efficiency, we implemented the Weighted Intersection over Union (WIoU) bounding box loss function (Cho, 2021; Tong et al., 2023) at the network’s output layer. The proposed method introduced an efficient IoU-based loss function that addressed the limitations of conventional approaches, achieving both accelerated convergence and enhanced regression accuracy. The WIoU loss function incorporated a dynamic non-monotonic focusing mechanism that more effectively evaluated anchor quality. This approach reduced dominance by high-quality anchors while mitigating harmful gradients from low-quality samples. This allowed the WIoU loss function to focus on anchor frames of ordinary quality and improve overall detection performance.

##### 2.2.3.1 SE attention mechanism

**Figure 6:**
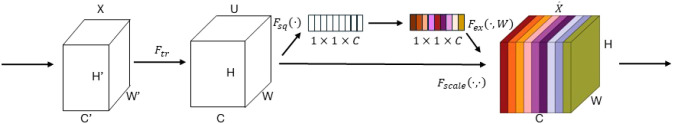
The structure of SE attention mechanism.

In the traditional convolutional neural network (CNN) architecture, convolutional layers and pooling layers were the core components for building deep feature representations (Sawarkar et al., 2024). These layers formed hierarchical feature representations by gradually extracting local features in the image and reducing the spatial dimension of the data. However, there was an implicit assumption in this process. That was, each channel of the feature map was equally important to the final task. Nevertheless, in practical applications, different channels often carry different amounts of information or importance, and contribute differently to the task (Chen et al., 2017). To solve this problem, Hu et al. proposed the SE attention mechanism architecture, which improved the model’s ability to express features by adaptively recalibrating the importance of each channel (Hu et al., 2018). Firstly, global average pooling was used to capture the global information of each channel, and then a channel weight vector was generated using a fully connected layer and a sigmoid activation function. Then, this weight vector was applied to each channel of the original feature map, and the feature map was scaled by element-by-element multiplication between channels, thereby enhancing the feature representation of those important channels and weakening the influence of those irrelevant or redundant channels. This adaptive channel recalibration mechanism enabled the model to focus more on the features that contributed most to the task, thereby improving the performance and generalization ability of the network. Therefore, a three-layer SE attention mechanism was added to the backbone network of the original YOLOv7 model. The structure diagram of the SE attention mechanism is shown in **Figure 6**.

##### 2.2.3.2 WIoU loss function

The Generalized Intersection over Union (GIoU) loss function was used in the original YOLOv7 architecture (Bochkovskiy et al., 2020; Wang et al., 2023). In most cases, GIOU can calculate IoU at a wide level. When predicted boxes perfectly coincided with ground truth boxes, their intersection area equaled their individual areas. Also, their minimum bounding rectangles were also the same. In this case, the GIoU value saturated at 1, making it unable to distinguish subtle deviations in predicted box alignment. Under this condition, GIoU reduced to standard IoU. Furthermore, the GIoU loss function suffered from two key limitations: (a) higher computational overhead, and (b) slower convergence compared to more recent alternatives. To calculate GIoU, it was necessary to find the minimum bounding rectangle for each predicted box and the true box. This approach introduced significant computational overhead and adversely impacted training convergence, particularly when processing high-volume datasets or high-resolution imagery. To solve this problem, this paper adopted the Weighted Intersection over Union (WIoU) loss function in the improved network. WIoU incorporated a dynamic non-monotonic mechanism for bounding box regression that adaptively modulated gradient distributions based on overlap states, effectively mitigating both excessive and harmful gradients form outlier samples. Through optimized gradient allocation, the WIoU loss function enhanced model performance in normal cases while demonstrating superior robustness in extreme scenarios, simultaneously accelerating convergence and improving training efficiency. Consequently, this study replaced the original YOLOv7’s GIoU loss function with the WIoU variant to enhance bounding box regression performance. The calculation process of GIoU and WIoU loss function were shown in the following Equations 1–6.


(1) $$I\text{o}U=\frac{\left|B_{p}\cap B_{gt}\right|}{\left|B_{p}\cup B_{gt}\right|}$$

(2) $$GI\text{o}U=IoU-\frac{\left|C\;\backslash \left(B_{P}\cup B_{gt}\right)\right|}{\left|C\right|}$$

(3) $$L_{GI\text{o}U}=1-GIoU$$

(4) $$\beta =\frac{\nu }{\alpha }.\frac{\left|B_{\text{p}}\cap B_{gt}\right|}{\left|B_{\text{p}}\cup B_{gt}\right|}$$

(5) WIoU=IoU·β

(6) $$L_{WI\text{o}U}=1-WIoU$$

Where *B_p_* is the predicted bounding box, *B_gt_* is the true bounding box, *C* is the smallest rectangle that can contain both the predicted box *B_p_* and the true box *B_gt_*, *β* is dynamic weight, *ν* is a normalization factor and *α* is a learnable parameter.

### 2.3 Experimental environment

The experiment platform in this research was an autonomously configured server running in the deep learning framework. The hardware environment is Intel Core i9-14900KF processor with 64GB running memory, and the graphics card is Nvidia GeForce RTX 4090 with 24GB memory. The software environment is a virtual environment built using Anaconda under the Ubuntu20.04 operating system. The virtual environment consisted of Pytorch 2.1.0, CUDA 12.3, and Python 3.8.6. The Python language was used as the main language for writing program codes. Also, the numpy, pandas, OpenCV and other required libraries were called to implement the training and testing of the lucky bamboo node detection model. The hardware and software configurations for the established model were listed in **Table 1**.

**Table 1:** The hardware and software configurations for the established model.

Project	Content
Operating system	Ubuntu20.04
CPU	Intel Core i9-14900KF
GPU	NVIDIA GeForce RTX 4090
RAM	64GB
Compiled language	Python3.8.6
Deep learning framework	CUDA12.3 Pytorch 2.1.0

### 2.4 Evaluation of bamboo node detection model

In this study, objective indicators such as precision, recall, and loss function convergence curve would be used to evaluate the performance of bamboo node detection model. Among them, Intersection over Union (IOU) represents the ratio of the intersection and union between the detected bounding box and the real bounding box, which is a common indicator for evaluating the performance of object detection model. The higher the IOU value, the better the model detection performance. Precision refers to the ratio of the number of correctly detected bamboo nodes to the total number of detected bamboo nodes. Recall is the ratio of the number of correctly detected bamboo nodes to the number of actual true bamboo nodes. The precision and recall were computed by Equations 7 and 8. True Positive (*TP*) indicates the number of correctly detected lucky bamboo nodes when the IOU is greater than or equal to the selected threshold. False Positive (FP) indicates the number of misjudged bamboo nodes when the IOU is smaller than the selected threshold. False Negative (*FN*) represents the number of undetected bamboo nodes. Average Precision (*AP*) refers to the area under the Precision-Recall curve. The higher the *AP* value, the better the performance of the model in detecting bamboo nodes. Mean Average Precision (*mAP*) refers to the mean value of *AP* for all categories. The AP and *mAP* were computed by Equations 9 and 10.


(7) $$P=\frac{TP}{TP+FP}$$

(8) $$R=\frac{TP}{TP+FN}$$

(9) $$AP=\int_{0}^{1}P\left(R\right)dR$$

(10) $$mAP=\frac{1}{N}\sum_{1}^{N}AP_{i}$$

## 3 Results and discussion

### 3.1 Performance of lucky bamboo node detection model

**Figure 7:**
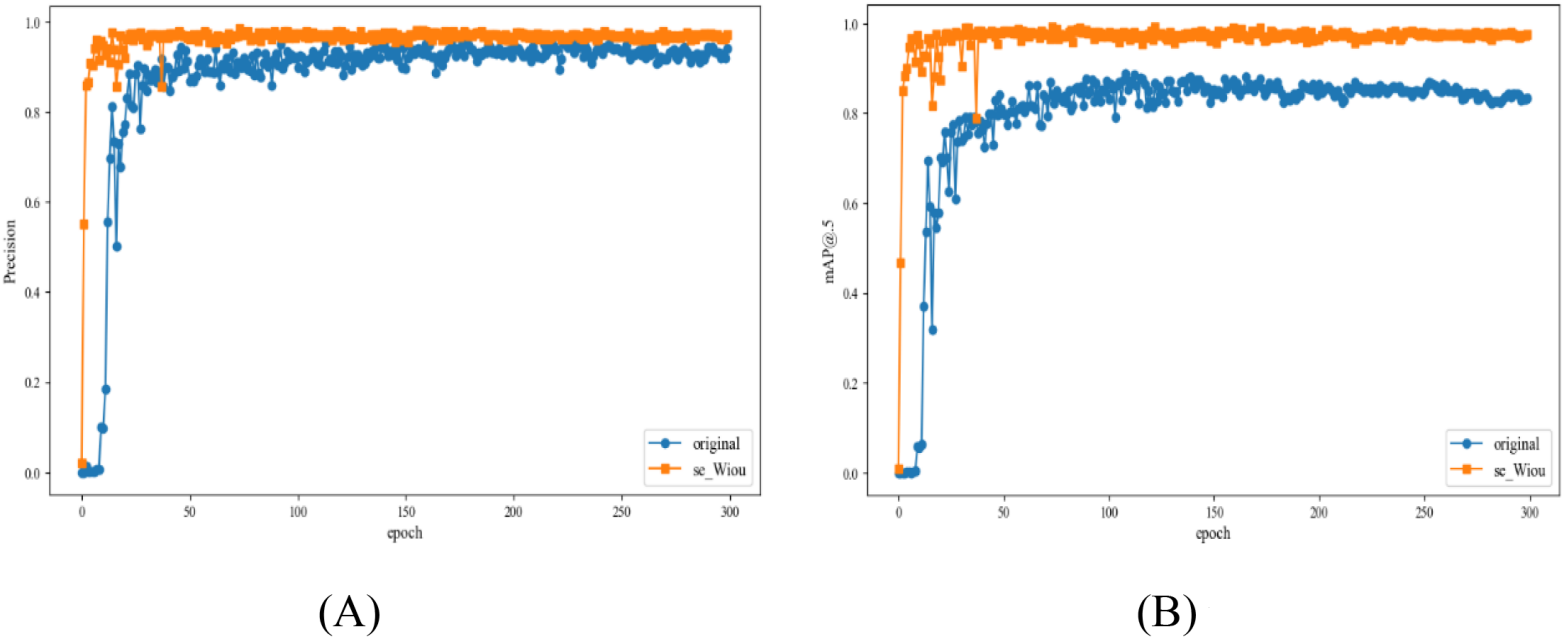
Comparison of training results between the original model and the lucky bamboo node detection model. **(A)** precision curve, **(B)** mAP curve.

The training results of lucky bamboo node detection model are shown in **Figure 7**. With the increase of training epochs, the precision value, and mAP value of lucky bamboo node detection model gradually increased. The details are as follows. During epochs 0-20, the precision values of the model increased rapidly. Afterwards, the precision values of the model remained stable between 0.95 and 0.97. Similarly, the mAP of the model increased rapidly during epochs 0–20 and then remained stable between 0.96 and 0.99. Conclusively, the training was suspended at 300 epochs.

### 3.2 Detection result of lucky bamboo node detection model

**Figure 8:**
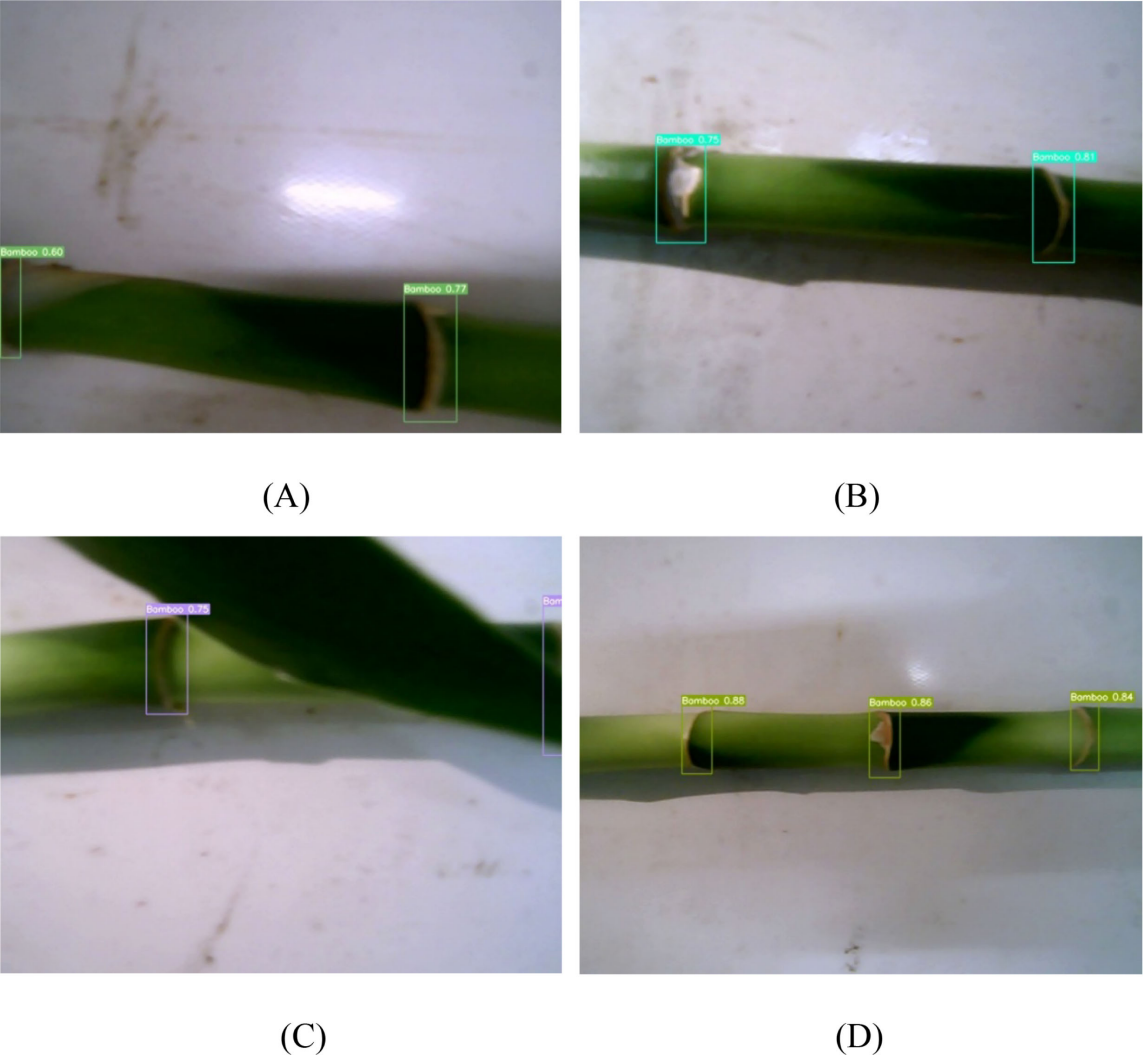
Detection results of lucky bamboo node detection model. **(A)** low light condition, **(B)** high light condition, **(C)** complex condition, **(D)** 10 cm shooting distance.

The lucky bamboo node detection model was tested on additional 400 RGB images collected later. **Figure 8** shows some examples of the detected bamboo nodes in different environmental conditions. The confidence scores were indicated beside each detected node. The high scores (up to 1.000) demonstrated that the results were quite reliable. In **Figure 8A**, even though the lucky bamboo plant was in low-light environment and blurry, the bamboo nodes thereon can still be correctly detected. In images taken in high-light conditions, the bamboo nodes appeared brighter and the bamboo nodes were similar in color to the background and surrounded by black shadows. It would be hard to recognize them manually, but the developed model was able to detect all the nodes in the image with high confident scores (**Figure 8B**). In **Figure 8C**, the bamboo nodes were occluded by a bamboo leaf, and one of them was occluded by more than 50%. But they were all successfully detected. In images taken at a high shooting distance condition (**Figure 8D**), the bamboo nodes accounted for a small proportion of pixels in the image, which would be difficult to be recognized. Nevertheless, every bamboo node was recognized with a high confidence score.

**Figure 9:**
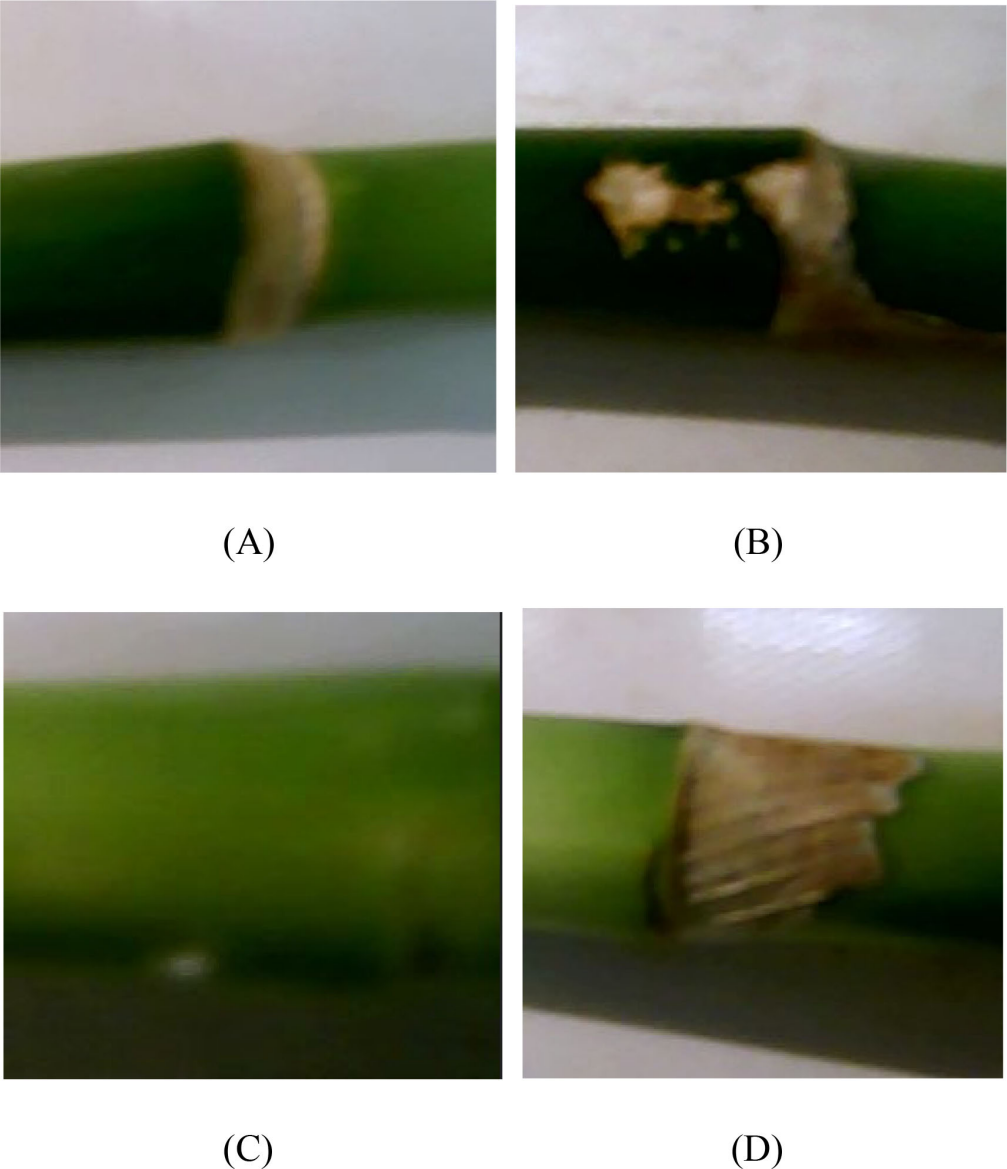
Examples of images which might have caused bamboo nodes detection errors. **(A)** blurred conditions, **(B)** similarities of bamboo spot and node, **(C)** sick bamboo node, **(D)** dried bamboo leaf.

Among the 400 images, a total of 1385 bamboo nodes were manually counted from the captured images by two people. With the same 400 images, the lucky bamboo node detection model detected 1376 true positives, 84 false positives, and 9 false negatives. Comparing two sets of results, the lucky bamboo node detection model was in good agreement with the manual counting, as indicated by the low errors, for example, 0.346 for RMSE. The minor discrepancies between the lucky bamboo node detection model and the manual counting could attribute to the following reasons. When training the CNN model, the white mottled rings of lucky bamboo (**Figure 9A**), the blocky mottled patches (**Figure 9B**), and the blurred bamboo body with scratches (**Figure 9C**) were not labeled when manually annotating the bamboo nodes. This meant that the model was not trained for these cases. This may lead to false positives (FP) in the detection. Avoiding these cases would improve the accuracy of bamboo node detection. In some cases, dry bamboo leaves that were not completely removed severely blocked the bamboo nodes, causing the CNN model to mistakenly classify the nearby nodes as background (**Figure 9D**). All these would explain the higher nodes counts of the bamboo node detection model. The resulting discrepancies could be minimized by labeling white mottled rings, blocky mottled patches, and blurred bamboo body with scratches as additional categories during image preprocessing, which would be explored in future studies. Overall, the low error showed that the model could successfully detect most of the bamboo nodes under various environmental conditions.

### 3.3 Ablation experiment

To further verify that the bamboo node detection model with added SE attention mechanism and WIoU loss function performs better than the original YOLOv7 model, four ablation experiments were conducted. The same data set and the same training parameters and methods were used to complete the training of each set of experiments. The experimental results are shown in **Table 2**, where “×” represents that the corresponding improvement strategy is not used in the network model, and “√” represents that the improvement strategy is used. Among them, Model 1 is the original YOLOv7 model, and model 2, model 3 and model 4 are models that add the SE attention mechanism, WIoU loss function, and SE attention mechanism-WIoU loss function to the original YOLOv7 model, respectively. The results showed that the detection accuracy (mAP) of the model 1 without any improvement strategy was 83.4%. Model 2, which introduced the SE attention mechanism based on the original YOLOv7 model, embedded spatial position information into channel attention. This enabled the model to achieve better prediction results when detecting bamboo nodes that relied on position information, thereby improving the detection accuracy (mAP) by 15.5%. Model 3 introduced a new bounding box loss function MIoU to reduce the overlap error between the predicted box and the true box, thereby improving the detection accuracy and stability of the model. Therefore, the detection accuracy (mAP) of model3 was improved by 12.7%. Model 4 (i.e. our developed model), which introduced the SE attention mechanism and MIoU loss function based on the original model, optimized the prediction accuracy of the YOLOv7 model for the recognition and localization of bamboo nodes, thereby improving the model detection accuracy (mAP) by 14.2%. Over all the four pretrained models, the model 4 had the best mAP (97.6%).

**Table 2:** Results of ablation experiment.

Model name	Improvement strategies		mAP (%)
	SE-mechanism	WIoU	
Model 1	×	×	83.4
Model 2	✓	×	98.9
Model 3	×	✓	96.1
Proposed model	✓	✓	97.6

**Figure 10:**
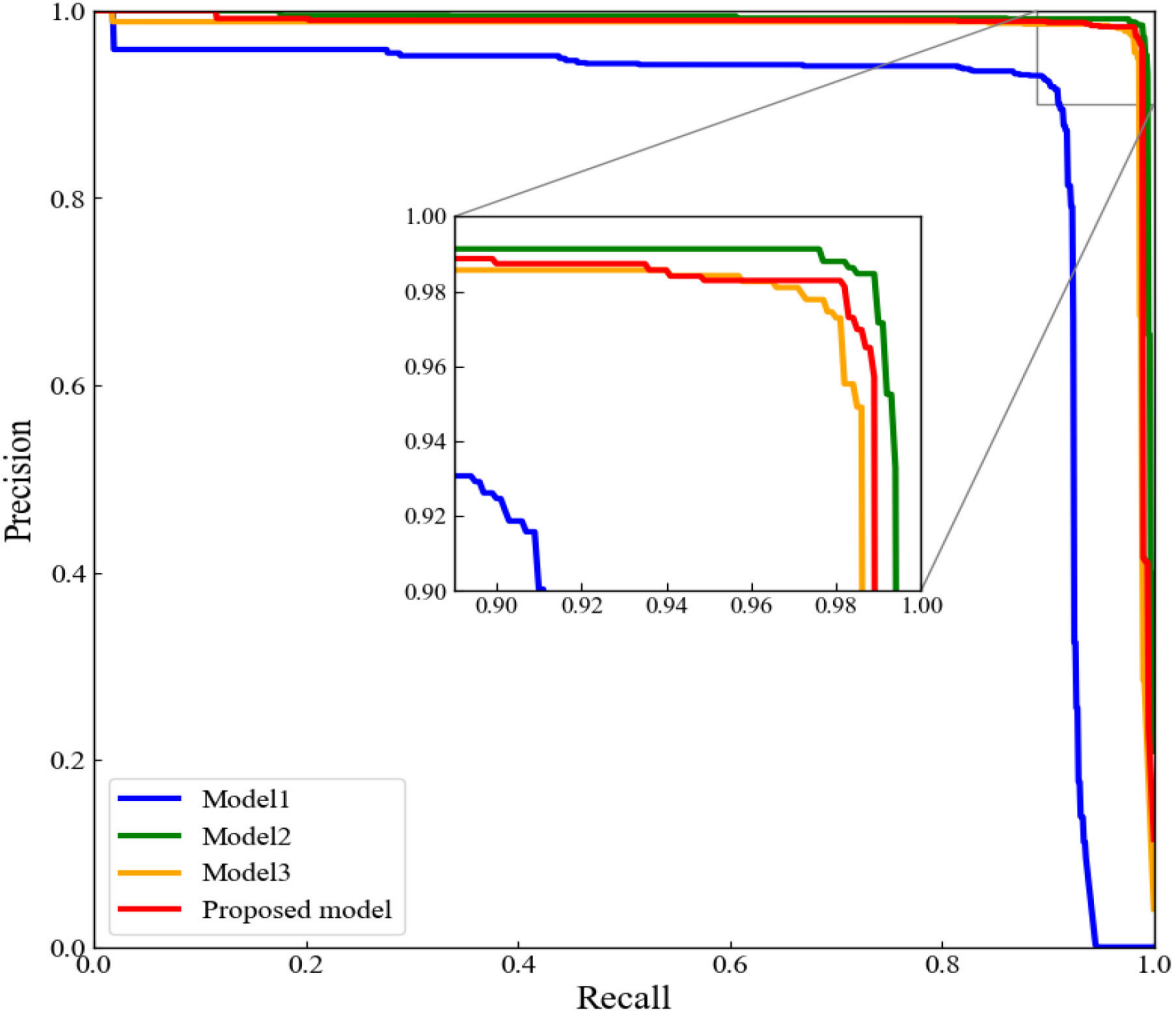
P-R curve of ablation experiment.

To further evaluate the improved models, the P-R curves for the four models were plotted. The P-R curves showed that the model 4 had the highest precision consistently over the recall range of 0.90 to 1.00 (**Figure 10**). All these performance indicators proved that our proposed lucky bamboo node detection model was superior than the other three models.

### 3.4 Comparison with other latest models

To better demonstrate the excellent performance of the proposed model, the proposed model was compared with the current mainstream YOLOv11 and YOLOv12 models. Compared to the baseline YOLOv7, our proposed model demonstrated comparable inference speed but achieved a significant 17.03% improvement in mAP (0.976). When benchmarked against Model 2, YOLOv11, and YOLOv12, our proposed model showed moderate mAP reductions of 1.3%, 1.22%, and 0.41% respectively. However, it delivered substantial Frame Per Second (FPS) enhancements-increasing FPS from 75.20 to 100.18 against Model 2, 70.8 to 100.18 versus YOLOv11, and 39.54 to 100.18 compared to YOLOv12 (**Table 3**). These computational efficiency gains represent critical advantages for industrial deployment scenarios. Consequently, our proposed model exhibited reduced inference time and higher processing throughput, demonstrating particular advantages for video stream analysis and enhanced suitability for real-world deployment scenarios.

**Table 3:** Comparison results with other latest models.

Model name	Parameter quantity (MB)	Average inference time (ms)	Frames Per Second (FPS)	mAP@0.5 (%)
**Proposed model**	136.04	**9.98**	**100.18**	0.976
YOLOv7_original	135.52	9.98	100.18	0.834
Model 2	135.52	13.30	75.20	**0.989**
YOLOv11	**109.11**	14.12	70.8	0.988
YOLOv12	227.44	25.29	39.54	0.980

Bold values indicate the best performance achieved in each column (i.e., lowest parameter quantity, fastest inference time, highest FPS, and highest mAP@0.5).

## 4 Conclusion

In this study, a high-precision and high-efficiency method was proposed based on a deep learning CNN mode for automatic detection of lucky bamboo node on the bamboo plant. Using the method, a high-throughput and low-cost application was developed and evaluated using lucky bamboo plant samples. The following conclusions were drawn. The CNN-based lucky bamboo node detection model was capable of recognizing and locating bamboo node in the lucky bamboo plant. The model was found to be the most efficient model for recognizing and locating bamboo nodes on the lucky bamboo plant structure. When compared to manual detection of bamboo node, the developed method had an estimated accuracy of 97.6%. The accuracy of the developed method was not affected by complex environment. The developed method shows great promise as a robust tool for computer-aided detecting of bamboo nodes on the lucky bamboo plant structure, which will help artisans rapidly and accurately identify lucky bamboo nodes to speed up the processing of lucky bamboo. However, more tests may be required to further verify the developed method.

## Data Availability

The raw data supporting the conclusions of this article will be made available by the authors, without undue reservation.
